# Epiglottic Epidermoid Cyst: Cadaveric Case Report and Clinical-Surgical Applications

**DOI:** 10.7759/cureus.38539

**Published:** 2023-05-04

**Authors:** Juan J Cardona, Katie Stormes, Neal Jackson, Yushi Abe, Joe Iwanaga, R. Shane Tubbs

**Affiliations:** 1 Department of Neurosurgery, Tulane Center for Clinical Neurosciences, Tulane University School of Medicine, New Orleans, USA; 2 Department of Otolaryngology, Tulane University School of Medicine, New Orleans, USA; 3 Department of Neurosurgery, Tulane University School of Medicine, New Orleans, USA; 4 Dental and Oral Medical Center, Kurume University School of Medicine, Kurume, JPN; 5 Department of Neurology, Tulane Center for Clinical Neurosciences, Tulane University School of Medicine, New Orleans, USA; 6 Department of Oral and Maxillofacial Anatomy, Graduate School of Medical and Dental Sciences, Tokyo Medical and Dental University, Tokyo, JPN; 7 Department of Anatomical Sciences, St. George's University, St. George's, GRD; 8 Department of Structural and Cellular Biology, Tulane University School of Medicine, New Orleans, USA; 9 Department of Surgery, Tulane University School of Medicine, New Orleans, USA; 10 Neurosurgery and Ochsner Neuroscience Institute, Ochsner Health System, New Orleans, USA

**Keywords:** anatomy, clinical, difficult airway, epiglottis, epidermoid cyst

## Abstract

An epidermoid cyst is lined with stratified squamous epithelium with a lumen filled with fluid, in most cases. Such cysts can occur anywhere in the body; however, they are rarely found on the epiglottis (0.54%). Herein, we describe to our knowledge, the first cadaveric case of a regular, circular, and soft mass extending out from the tip of the epiglottis with consistent histological characteristics of an epidermoid cyst. Epiglottic cysts are rare and mostly asymptomatic. However, through this case report, we aimed to highlight the clinical-surgical applications presented mainly when they grow large enough, to cause issues with ventilation or obstruct endotracheal tubes, thus interfering with airway management. Additionally, such cysts can affect swallowing or speaking.

## Introduction

An epidermoid cyst is defined as a “simple cyst lined with stratified squamous epithelium and a lumen filled with cystic fluid or keratin and no other specialized structure” [1]. This type of cyst can be found anywhere in the body with an incidence of 7% in the head and neck regions, most commonly the face, neck, and periauricular areas [2]. The estimated incidence of cysts involving the epiglottis is 0.54%, they can vary in size between 1 and 5 cm, being rarer to find epiglottic cysts with bigger sizes (0.02%) during laryngoscopy [2,3].

These cysts tend to grow slowly and are asymptomatic in most cases. Depending on their size and location, they can potentially cause symptoms such as dyspnea, dysphagia, hoarseness, foreign body sensation, and airway obstruction [4,5]. They are often discovered incidentally during screening endoscopy or intubation. In these circumstances, they are associated with complications such as difficult intubation and ventilation as well as cyst rupture [3,6]. This report describes the first, to our knowledge, cadaveric case of an epiglottic epidermoid cyst and considers the clinical and surgical implications of the relationship with its place of development, size, orientation, and its adjacent structures.

## Case presentation

**Figure 1:**
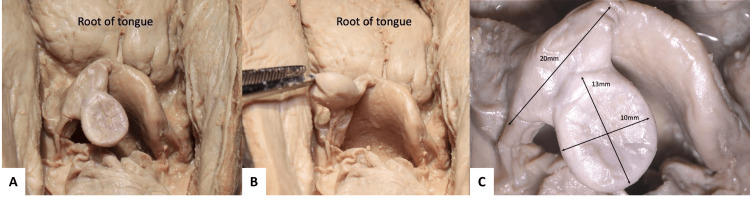
A large mass identified on the epiglottis (posterosuperior views). The posterior wall of the pharynx is incised. (A) Neutral position of the mass. (B) The mass turned anteriorly. (C) Magnified image of the mass with measurement results.

During the routine dissection of the laryngeal region in a fresh-frozen 64-year-old at-death Caucasic male cadaver, a mass was identified on the epiglottis. The width of the mass at its root was 20 mm (Figures 1A-1C).

**Figure 2:**
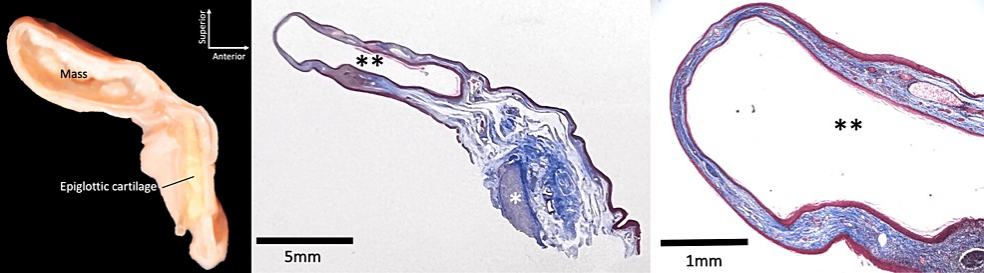
Left: The epiglottis along with the mass was incised sagittally. Middle: The mass was filled with keratinized tissue. Right: Stratified squamous epithelium lining the epiglottis and the lumen of the cyst. *epiglottic cartilage. **lumen of the cyst.

This mass had a regular and circular shape, with a soft consistency and was apparently filled with keratinized tissue. It extended out from the wall of the tip of the epiglottis and was oriented posteriorly toward the laryngopharynx obstructing the airway and the esophagus. Next, the epiglottis along with the mass was incised sagittally and examined histologically using Masson’s Trichrome staining. This analysis revealed stratified squamous epithelium lining the epiglottis and the lumen of the cyst (Figure 2). These findings were consistent with an epidermoid cyst.

For this report, the authors state that every effort was made to follow all local and international ethical guidelines and laws that pertain to the use of human cadaveric donors in anatomical research [7].

## Discussion

Epidermoid cyst characteristics

An epidermoid cyst is also known as an infundibular cyst, epidermal cyst, epidermal inclusion cyst, and epidermoid inclusion cyst. This type of cyst occurs more frequently in males, at any age, as either solid or cystic, solitary or multiple, small or large masses, and mostly is benign with only approximately 1% of cases having a malignant transformation to squamous cell carcinoma or basal cell carcinoma [2].

Epidermoid cysts are commonly confused with dermoid cysts. They differ from dermoid cysts because of the lack of skin appendages. They are diagnosed mainly in adulthood, and dermoid cysts commonly are located superficially or in the anterior orbit; however, epidermoid cysts can grow anywhere in the body, they are rarely found in the oral cavity and even more infrequently at the level of the hypopharynx at the epiglottis, as found in our study [2].

Ultrasound is commonly used for the evaluation of palpable soft-tissue masses such as epidermoid cysts. They have the usual appearance of cysts although sometimes may present as cystic to solid masses. When other diagnostic images are needed, a CT scan is useful mainly for planning the intervention and diagnosing patients with large-size cysts [2,4].

Origin and pathophysiology

Epidermoid cysts are categorized as acquired or congenital. There are a few theories concerning the development of these cysts, described by the following case reports. Hoang et al. [2] stated they can grow spontaneously or due to implantation of the epithelium secondary to injuries. On the other hand, Nishar et al. [5] in a case report of a 60-year-old male with an unexpected giant sublingual epidermoid cyst, discussed that during the third and fourth embryonic weeks, epidermoid cysts can develop from the entrapment of ectodermal elements at the fusion sites of the first and second pharyngeal arches. Moreover, Chida et al. [4], in a case report of a 77-year-old male with a giant epiglottic cyst, described that the development of laryngeal cysts is related to chronic mucosal inflammation with subsequent obstruction and dilation of mucus ducts.

Clinical-surgical implications

Laryngeal cysts grow slowly, and they are asymptomatic in most cases. Depending on their location, size, and orientation, such cysts can potentially cause symptoms, such as dyspnea, dysphagia, and foreign body sensation. However, when they are located at the epiglottis or vocal folds, they can cause hoarseness, dysphonia, and even airway obstruction.

Interestingly, Hou et al. [8] described a report of a 53-year-old male with dysarthria who underwent CT, MRI, and MRA of the brain to evaluate for cerebral infarction. When these imaging studies were negative, but his symptoms were still present, the patient underwent a CT of the neck that identified an epiglottic cyst that was causing his symptoms.

Similarly, Lee et al. [9] conducted a study to confirm the relationship between the anatomical change and resonance function of the vocal tract in eight males with epiglottic cysts before and after surgery. They found that the first formant value of the vowel /a:/ was significantly raised after surgery. However, changes in other acoustic parameters such as fundamental frequency, jitter, shimmer, noise-to-harmonic ratio, and second formant were not found to be significant. These alterations are very important for clinicians owing to their correlation with the health status of the laryngeal or pharyngeal regions, and the potentially dangerous situation of airway obstruction or suffocation.

On the other hand, cysts in the laryngeal region are a challenge for anesthesiologists and surgeons owing to the complexity of airway management associated with the presence of the cyst. Takaishi et al. [3] described a case series of six of 1,112 patients with a mean age of 66.2 years with an epiglottic cyst that was discovered incidentally during intubation. The authors recommend that a video laryngoscope is effective for assistance in an unexpectedly difficult airway with issues with ventilation and intubation. For their part, Zheng et al. [10] in a 46-day-old male infant with a large epiglottic cyst, recommends aspiration as the first-choice procedure for airway management, however, it is very important to consider the risk of infection and pulmonary aspiration associated with this intervention.

Declaration

The authors sincerely thank those who donated their bodies to science so that anatomical research could be performed. Results from such research can potentially increase mankind’s overall knowledge that can then improve patient care. Therefore, these donors and their families deserve our highest gratitude [11].

## Conclusions

Cysts affecting the epiglottis are rare. Although often asymptomatic, knowledge of the clinical-surgical applications when such cysts grow to a large size is important as the airway can be compromised. The treatment for such large epiglottic cysts is presented herein. To our knowledge, this is the first case report of this entity described in a cadaveric specimen.
